# Adipose Overexpression of Heme Oxygenase-1 Does Not Protect against High Fat Diet-Induced Insulin Resistance in Mice

**DOI:** 10.1371/journal.pone.0055369

**Published:** 2013-02-04

**Authors:** Jun-Yuan Huang, Ming-Tsai Chiang, Lee-Young Chau

**Affiliations:** 1 Institute of Biomedical Sciences, Academia Sinica, Taipei, Taiwan, Republic of China; 2 Graduate Institute of Life Sciences, National Defense Medical Center, Taipei, Taiwan, Republic of China; McGill University, Canada

## Abstract

Heme oxygenase-1 (HO-1) is a stress-responsive enzyme with potent anti-oxidant and anti-inflammatory activities. Previous studies have shown that systemic induction of HO-1 by chemical inducers reduces adiposity and improves insulin sensitivity. To dissect the specific function of HO-1 in adipose tissue, we generated transgenic mice with adipose HO-1 overexpression using the adipocyte-specific aP2 promoter. The transgenic (Tg) mice exhibit similar metabolic phenotype as wild type (WT) control under chow-fed condition. High fat diet (HFD) challenge significantly increased the body weights of WT and Tg mice to a similar extent. Likewise, HFD-induced glucose intolerance and insulin resistance were not much different between WT and Tg mice. Analysis of the adipose tissue gene expression revealed that the mRNA levels of adiponectin and interleukin-10 were significantly higher in chow diet-fed Tg mice as compared to WT counterparts, whereas HFD induced downregulation of adiponectin gene expression in both Tg and WT mice to a similar level. HFD-induced proinflammatory cytokine expression in adipose tissues were comparable between WT and transgenic mice. Nevertheless, immunohistochemistry and gene expression analysis showed that the number of infiltrating macrophages with preferential expression of M2 markers was significantly higher in the adipose tissue of obese Tg mice than WT mice. Further experiment demonstrated that myeloid cells from Tg mice expressed higher level of HO-1 and exhibited greater migration response toward chemoattractant in vitro. Collectively, these data indicate that HO-1 overexpression in adipocytes does not protect against HFD-induced obesity and the development of insulin resistance in mice.

## Introduction

Adipose tissue is a primary site in the body to store energy in the form of triglyceride [1] When dietary energy intake persistently exceeds energy expenditure, the adipose tissue can expand through hypertrophy of the existing adipocytes and generation of new adipocytes, leading to the development of obesity [2]. Obesity caused by the sedentary life style and Western diet has become a prevalent health problem associated with increased incidence of insulin resistance, which is a major risk factor for type II diabetes and cardiovascular diseases [3]. Substantial works have revealed that obesity is associated with systemic oxidative stress and low-grade inflammation [4]–[5]. Adipocytes express a number of proinflammatory cytokines, including tumor necrosis factor-α (TNF-α), interleukin-6 (IL-6), and monocyte chemotactic protein-1 (MCP-1), which are upregulated in the adipose tissues of obese subjects [6]. In contrast, the expression of adiponectin, the adipocyte-derived adipokine with potent function in regulating insulin sensitivity, is downregulated during obesity [6]. In parallel, macrophage infiltration is increased in the adipose tissues and contributes to the adipose inflammation and the development of insulin resistance in obesity. Moreover, the adipose tissue macrophages have been shown to exhibit in two different phenotypes, the classically activated M1 or alternatively activated M2 macrophages [7]–[9]. The resident macrophages in lean adipose tissues are primarily in M2 state, which expresses immunosuppressive interleukin-10 (IL-10) but downregulates inducible nitric oxide synthase (iNOS) [7]–[9]. Obesity promotes adipose macrophage accumulation with a phenotypic switch to M1 phenotype expressing CD11c and proinflammatory cytokines [7]–[9].

Heme oxygenase-1 (HO-1) is a stress-inducible enzyme catalyzing the oxidative degradation of heme to release free iron, carbon monoxide (CO), and biliverdin [10]. In addition to its primary role in heme catabolism, numerous studies have supported the vital function of HO-1 in various pathophysiological states associated with cellular stress. It has been shown that HO-1 protects cardiovascular system against various insults by virtue of the anti-oxidant properties of the biliverdin and its metabolite, bilirubin, and the anti-inflammatory effect of CO, suggesting that HO-1 is a potential therapeutics for cardiovascular diseases [10]. HO-1 has been shown to highly express in the white adipose tissue (WAT) of genetic and high fat-diet (HFD)-induced obese mice [11]–[12]. However, the pathophysiological role of adipose HO-1 during obesity and the development of insulin resistance has not yet been fully characterized. Over the past few years, there were studies showing that systemic induction of HO-1 by treatment with HO-1 inducer, hemin or cobalt protoporphyrin, in ob/ob mice or Zucker diabetic rats reduced adiposity and improved insulin sensitivity [13]–[15]. The protective effect of systemic HO-1 induction was attributed to an increase in adiponectin expression, enhanced AMP kinase activation in both adipocytes and skeletal muscles, and suppression of adipogenesis and inflammatory cytokine expression. Nevertheless, a study has shown that the endogenous HO-derived CO was increased and promoted hypertension and endothelial dysfunction in obese Zucker rats [16]. More recently, a study from our group also demonstrated that hematopoietic HO-1 expression promoted macrophage infiltration in adipose tissue and the development of insulin resistance [12], indicating that HO-1 may impact this complicated disease through its differential effects on various cell compartments. To dissect the potential discrete roles of HO-1 in different cell types implicated in the metabolic disease, here we generated transgenic mice overexpressing HO-1 in adipocytes to study the effect of adipocyte HO-1 on diet-induced adiposity and insulin resistance.

## Materials and Methods

### Ethics Statement

All experimental procedures with animals were approved by the Institutional Animal Care and Utilization Committee of the Academia Sinica, Taiwan (Protocol #: RMiIBMCL2008070).

### Animals

DNA fragment encoding human full length HO-1 was amplified by the polymerase chain reaction (PCR) from pAdCMV-HO-1 [17] and subcloned into mouse pBS aP2 promoter polyA plasmid (Addgene). The transgene consisting of 5.4 kb of the aP2 gene promoter, the human HO-1 cDNA and a polyadenylation sequence was cut from the vector with ClaI and SacII restriction enzymes and subjected to pronuclear microinjection. The HO-1 transgenic (Tg) mice in C57BL/6J genetic background were generated by Level Biotechnology (New Taipei City, Taiwan R.O.C). HO-1 Tg founders were then bred to generate F1 Tg mice and subjected to Southern blot and PCR analysis to check the transgene expression. The Tg F2 offspring was generated by crossing the F1 Tg mice with wild type (WT) C57BL/6J mice and their descendants were used in the experiments. In diet-induced obesity, 8 weeks old WT and HO-1 Tg male mice were divided into two groups and fed either a regular chow (10 kcal% fat, 5058, LabDiet) or a high fat diet (60 kcal% fat, D12492, Research Diets) for 12 weeks. All mice were kept on a 12 h light-dark cycle and allowed free access to food and water.

### Isolation of Genomic DNA and Genotyping

Mouse tail snip (∼0.5 cm) was incubated in 700 µl of lysis buffer containing 50 mM Tris-HCl pH 8.0, 100 mM EDTA, 0.5% SDS and 360 µg proteinase K at 55°C for 18 h. Samples were then mixed with 700 µl of phenol/chloroform/isoamyl alcohol (25∶24:1), vortexed for 3 min at room temperature, and centrifuged at 15,500×g for10 min at 4°C. The upper layer was transferred to a new tube, mixed with equal volume of chloroform/isoamyl alcohol (24∶1) and vortexed for 3 min at room temperature. Then the sample was centrifuged at 15,500×g for 10 min at 4°C, and the upper layer was removed and mixed with equal volume of isopropanol. After gently mixing, the genomic DNA was pelleted by centrifugation at 15,500×g for 10 min, rinsed once with 75% alcohol, air dried, and resuspended in 100 µl ddH_2_O. Genotyping PCR was performed by using 50 ng genomic DNA, 62.5 pmole primer, 10 nmole dNTPs and 2.5 U FastStart Taq DNA polymerase (Roche). The primer used were:5′ -primer, 5′-ATTGCCCAGAAAGCCCTGGACC-3′ and 3′- primer, 5′-GATCCGAATTCGCCCTATAG-3′. The PCR condition was 94°C 5 min; 35 cycles of 94°C 30 sec, 50°C 30 sec, and 72°C 30 sec; then 72°C 7 min.

### Southern Blot Analysis

Mouse genomic DNA (10 µg ) was digested with SacI at 37°C for 2 h. Copy number standards were prepared by mixing WT mice genomic DNA with known amount of transgene plasmid DNA followed by SacI digestion. Following 1% agarose gel electrophoresis, gel was soaked in denaturation buffer containing 0.5 N NaOH and 1.5 M NaCl for 40 min and then in neutralization buffer containing.5 M Tris-HCl, pH7.5 and 3 M NaCl for another 40 min. DNA was then transferred to Hybond-N+ membrane (GE Healthcare Life Sciences) by vacuum blotting system (VacuGene XL, Pharmacia LKB) for 5 hr. The DNA was fixed to membrane by UV crosslinking. Southern blot analysis was performed using Digoxigenin (DIG) High Prime DNA Labeling and Detection Starter Kit II (Roche) according to the manufacturer's instruction. To prepare the DIG-labeled DNA probe, 1 µg of a human HO-1 cDNA fragment (∼0.7 kb) was first denatured by boiling in water for 10 min and then cooled down on ice. The denatured DNA was incubated with 4 µl DIG-High Prime at 37°C for 20 h. The reaction was stopped by adding 2 µl of 0.2 M EDTA (pH 8.0) and heating to 65°C for 10 min. The labeling efficiency was determined by comparing with the serial diluted DIG-labeled control DNA. To perform the Southern blot analysis, the membrane was preincubated with 10 ml of pre-heat DIG Easy Hyb (Roche) at 42°C for 30 min in the hybridization bag. The DIG-labeled DNA probe specific for human HO-1 cDNA (25 ng/ml) was then added to the hybridization bag. After overnight incubation at 42°C, the membrane was washed twice with buffer containing 2× saline-sodium citrate buffer (SSC) and 0.1% SDS for 5 min at room temperature, followed by wash twice with buffer containing 0.5× SSC and 0.1% SDS for 15 min at 60°C. Subsequently, the membrane was rinsed once with buffer A (0.1 M maleic acid, pH 7.5, 0.15 M NaCl, and 0.3% Tween-20), and incubated with blocking solution (Roche) and then antibody solution (anti-DIG-AP conjugate, 1/10,000 in blocking solution) for 30 min at room temperature, respectively. After two washes with buffer A for 15 min/wash, membrane was equilibrated in detection buffer (0.1 M Tris-HCl pH 9.5 and 0.1 M NaCl) for 5 min. The chloro-5-substituted adamantyl-1,2-dioxetane phosphate was applied to the membrane and the luminescence signal was detected by X-ray film exposure.

### Metabolic Parameter Analysis

Blood glucose level was measured using automatic blood glucose meter (ACCU-CHEK Advantage, Roche, IN, USA). Serum insulin level was measured with mouse insulin ELISA kit (Millipore). Cholesterol and triglyceride concentrations were measured by FUJI DRI-CHEM clinical chemistry analyzer (FUJIFILM, Tokyo, Japan). Glucose tolerance test was performed by intraperitoneal (i.p.) injection of glucose (1g/kg of body weight) in mice fasted for 16 h. For insulin tolerance test, mice fasted for 6 h received i.p.injection of insulin (1.3 U/kg of body weight). Blood samples were drawn from the tail vein at the indicated time point and glucose concentration was measured with an automatic blood glucose meter.

### Peritoneal Macrophage Isolation

Mice received i.p. injection of 3 ml 3% thioglycollate. After 4 days, peritoneal macrophages were isolated by washing the peritoneal cavity with RPMI 1640 medium containing 0.5% bovine serum albumin (BSA). Cells were then plated on cell culture plate. After 4 h incubation, the nonadherent cells were removed by washing with culture medium and the adherent cells used as peritoneal macrophages.

### Real-time Quantitative PCR

Total RNA were isolated with TRIzol reagent. Reverse transcription was performed by using 2 µg of DNase I-treated total RNA, 100 ng random hexamers, 10 nmole dNTPs and 200 U Superscript III reverse transcriptase. Real-time quantitative PCR was performed with LightCycler FastStart DNA Master^PLUS^ SYBR Green I kit (Roche) on a LightCycler Carousel-Based System (Roche Applied Science, IN, USA). 36B4 was used as an internal control. The primer pairs were TNFα (5′-AGACCCTCACACTCAGA-3′ and 5′-CCTTGTCCCTTGAAGAGAAC-3′), IL-6 (5′-GAGGATACCACTCCCAACAGACC-3′ and 5′-AAGTGCATCATCGTTGTTCATACA-3′), vascular endothelial growth factor (VEGF) (5′-CAGGCTGCACCCACGACAGAAG-3′ and 5′-CTATGTGCTGGCTTTGGTGAGGTTT-3′), sromal cell-derived factor-1 (SDF-1) (5′-ATGGACGCCAAGGTCGTCGCC-3′ and 5′-TTACTTGTTTAAAGCTTTCTC-3′), MCP-1 (5′-CTTCTGGGCCTGCTGTTCA -3′ and 5′-CCAGCCTACTCATTGGGATCA-3′), adiponectin (5′-GCAGAGATG GCACTCCTGGA-3′ and 5′-CCCTTCAGCTCCTGTCATTCC-3′), F4/80 (5′-TTTCCTCGCCTGCTTCTTC-3′ and 5′-CCCCGTCTCTGTATTCAACC-3′), interleukin-10 (IL-10) (5′-GGTTGCCAAGCCTTATCGGA-3′ and 5′-ACCTGCTCCACTGCCTTG CT-3′), CD11c (5′-GAGAGCCCAGACGAAGACAG-3′ and 5′-CCATTTGCTTCCTCCAACAT-3′), Mannose receptor (MR) (5′-GCAAATGGAGCC GTCTGTGC-3′ and 5′-CTCGTGGATCTCCGTGACAC-3′), iNOS (5′- TGCATGGACCAGTATAAGGCAAGC-3′ and 5′- GCTTCTGGTCGATGTCATGAGCAA-3′), macrophage galactose-type C-type lectin 1 (Mgl1) (5′- TGAGAAAGGCTTTAAGAACTGGG-3′ and 5′- GACCACCTGTAGTGATGTGGG-3′) and 36B4 (5′-CCCACTTACTGAAAAGG-3′ and 5′-GGCGGGATTAGTCGAA-3′).

### Western Blot Analysis

Tissues or cells were lysed in buffer containing 50 mM Tris, pH 7.4, 150 mM NaCl, 1% Nonidet P-40, 0.25% sodium deoxycholate, 1 mM EDTA, 1 mM PMSF and 1% protease inhibitor, followed by centrifugation at 15,500×g for 10 min at 4°C. The tissue or cell lysates (supernatants) were collected and the protein concentrations determined. The protein samples were separated by electrophoresis in 10% SDS-polyacrylamide gel and transferred to Immobilon-P Membrane (Millipore). After blocking with 5% skim milk in Tris-buffered saline containing 0.1% Tween-20 (TBST), the membrane was incubated with rabbit anti-HO-1 antibody [18](1∶2000 dilution) or goat anti-Mgl1 antibody (R&D systems; 1∶1000 dilution) in TBST containing 1% skim milk for 2 h at room temperature. The membrane was then washed with TBST for three times and incubated with horseradish peroxidase (HRP)-conjugated anti-rabbit ((Santa Cruz; 1∶3000 dilution) or anti-goat (Santa Cruz; 1∶2000 dilution) secondary antibody in TBST containing 5% skim milk for another 1 h. After three washes with TBST, antigen-antibody complex was detected using Western Lightning Plus-ECL (PerkinElmer). To repobe the membrane with anti-GAPDH antibody (Santa Cruz; 1∶3000 dilution), the membrane was incubated in stripping buffer containing 62.5 mM Tris-HCl, pH 6.8, 2% SDS, and 100 mM 2-mercaptoethanol at 55°C for 30 min with shaking. After washing with TBST for three times, the membrane was blocked and incubated with antibody as described above. For immunoblot quantification, the X-film was scanned by ScanMaker 8700 (MICROTEK, Hsinchu, Taiwan) and images were analyzed using MetaMorph image analysis software. The band intensity of target protein was normalized by dividing its intensity value with the value of the internal control protein (GAPDH) band in the same lane of the blot. Data were presented as the normalized intensities of at least 3 mice in each group.

### HO Activity Measurement

Epididymal adipose tissue was homogenized in ice cold buffer containing 0.1 M K_3_PO_4_ pH 7.4, 1 mM EDTA and 0.5 mM PMSF, and centrifuged at 15,500×g for 10 min at 4°C. The supernatant was removed and 1 mg of proteins were incubated in 200 µl of buffer containing 0.1 M K_3_PO_4,_ pH 7.4, 1.5 mg of rat liver cytosol, 50 µM hemin, 1 mM NADPH, 2 mM glucose-6-phosphate and 0.3 units of glucose-6-phosphate dehydrogenase. The mixture was incubated in the dark for 1 h at 37°C. Bilirubin was then extracted with 1 ml chloroform and determined by the absorbance difference between 464 and 530 nm with an extinction coefficient of 40/mM*cm.

### ELISA

The adipokine levels in mouse serum samples and adipose tissue lysates were determined using the indicated ELISA kits (R&D Systems) according to the manufacturer’s instructions. For tissue adipokine levels, data were represented as the amount of cytokine per 100 µg of tissue lysates.

### Immunohistochemistry

Adipose tissue was fixed in Bouin’s solution for 16 h with shaking (30 rpm) at room temperature and embedded in paraffin. The 5 µm sections were deparaffinized in xylene and rehydrated. The endogenous peroxidase activity was depleted with 3% H_2_O_2_ for 10 min at room temperature. For immunostaining of F4/80, sections were first blocked with phosphate-buffered saline (PBS) containing 5% normal goat serum for 30 min at 37°C, followed by incubation with mouse anti-F4/80 antibody (eBioscience; 1∶300 dilution) in PBS for 1 h at 37°C. The sections were washed three times with PBS, and then incubated with HRP-conjugated goat anti-mouse (Santa Cruz; 1∶300 dilution) secondary antibody in PBS for 1 h at room temperature. After three washes with PBS, the sections were incubated with 0.1% 3,3′-diaminobenzidine (DAB) solution. The nuclei were counterstained with hematoxylin for 5 min at room temperature. The pictures were taken in four different fields per section (x 100) under a light microscope. The extent of macrophage infiltration was calculated as the percentage of adipocytes surrounded by a F4/80-positive crown-like structure in the total adipocytes in each field. For detection of Mgl1 expression, sections were blocked with PBS containing 5% donkey serum (Jackson ImmunoResearch), followed by incubation with goat anti-Mgl1 (R&D systems; 1∶100 dilution) in PBS for 1 h at 37°C. After 3 washes, the sections were incubated with HRP-conjugated donkey anti-goat (Santa Cruz; 1∶200) secondary antibody in PBS for 1 h at room temperature, washed, and incubated with 0.1% DAB for antigen detection. For immunostaining of the vasculature, sections were incubated with 20 µg/ml of proteinase K (Sigma-Aldrich) at room temperature for 5 min. After a PBS wash, the sections were blocked with 5% normal goat serum, followed by incubation overnight at 4°C with antibody against CD31 (BD Pharmingen;1∶100 dilution). After three PBS washes, the sections were incubated with FITC-conjugated secondary antibody (Invitrogen;1∶200 dilution) at room temperature for 1 h in the dark, followed by washing, and examined by fluorescence microscopy. Vascular density was calculated as described previously [12]. Briefly, the CD31 immunofluorescence images were taken from 4 different fields per section under a fluorescence microscope (x 200 magnification). MetaMorph image analysis software (Molecular Devices) was used for image analysis. The CD31^+^ area in each field was calculated as [(CD31^+^ signal – background (FITC-goat anti-rat IgG))/total pixels] × 0.6183 mm^2^ (the total real area under 200× magnification). The vascular density was defined as CD31^+^ area per field (×10^−6^/um^2^). The normalized vascular density was calculated by dividing the CD31^+^ area with the total number of adipocytes per area. The adipocyte density was calculated by counting the number of adipocytes per area (×10^−6^/um^2^).

### Isolation of CD11b^+^ Myeloid Cells

Mouse spleen was minced into pieces in 2 ml serum free RPMI medium containing 0.5% BSA. The clumps were further dispersed by gently pipetting up and down several times. The cell suspension was then passing through a 70 µm cell strainer into a 50 ml tube, followed by centrifugation at 400×g for 5 min. The cell pellet was subjected to red blood cell lysis in ammonium chloride solution (BD Pharm Lyse™) for 5 min at room temperature. After washing once with PBS containing 2% fetal bovine serum (FBS) (PBS/2% FBS), cells were spin down at 300×g for 5 min at 4°C, and resuspended at a density of 1×10^8^ cells/ml in PBS/2% FBS. CD11b^+^ cells were enriched using the EasySep mouse CD11b positive selection kit (18770, Stemcell Tech) following the manufacturer’s instruction, and confirmed by flow cytometry.

### In vitro Migration Assay

Cell migration was performed using 24 well Transwell with a pore size of 5 µm (Corning). Briefly, CD11b^+^ cells (1×10^5^) isolated from WT and HO-1 Tg mice were suspended in serum free RPMI 1640 medium containing 0.5% BSA and added into the upper chambers. The lower chambers were filled with medium supplemented with 10 ng/ml of MCP-1. To test the effect of HO-1 inhibition, tin protoporphyrin IX (SnPP) was added into both upper and lower chambers to a final concentration of 10 µM prior to the addition of MCP-1 to the lower chambers. After 4 h incubation, unmigrated cells were wiped off the upper side of the filter with a cotton swab. The migrated cells on the lower side were fixed with 4% paraformaldehyde for 10 min, stained with Giemsa for 2 h, and counted under light microscope.

### Statistical Analysis

Results were expressed as the means ±S.E. Differences between groups were examined for statistical significance using Student’s t test or ANOVA. A *P*-value < 0.05 was considered statistically significant.

## Results

### Generation of Transgenic Mice with Adipose Overexpression of Human HO-1

**Figure 1:**
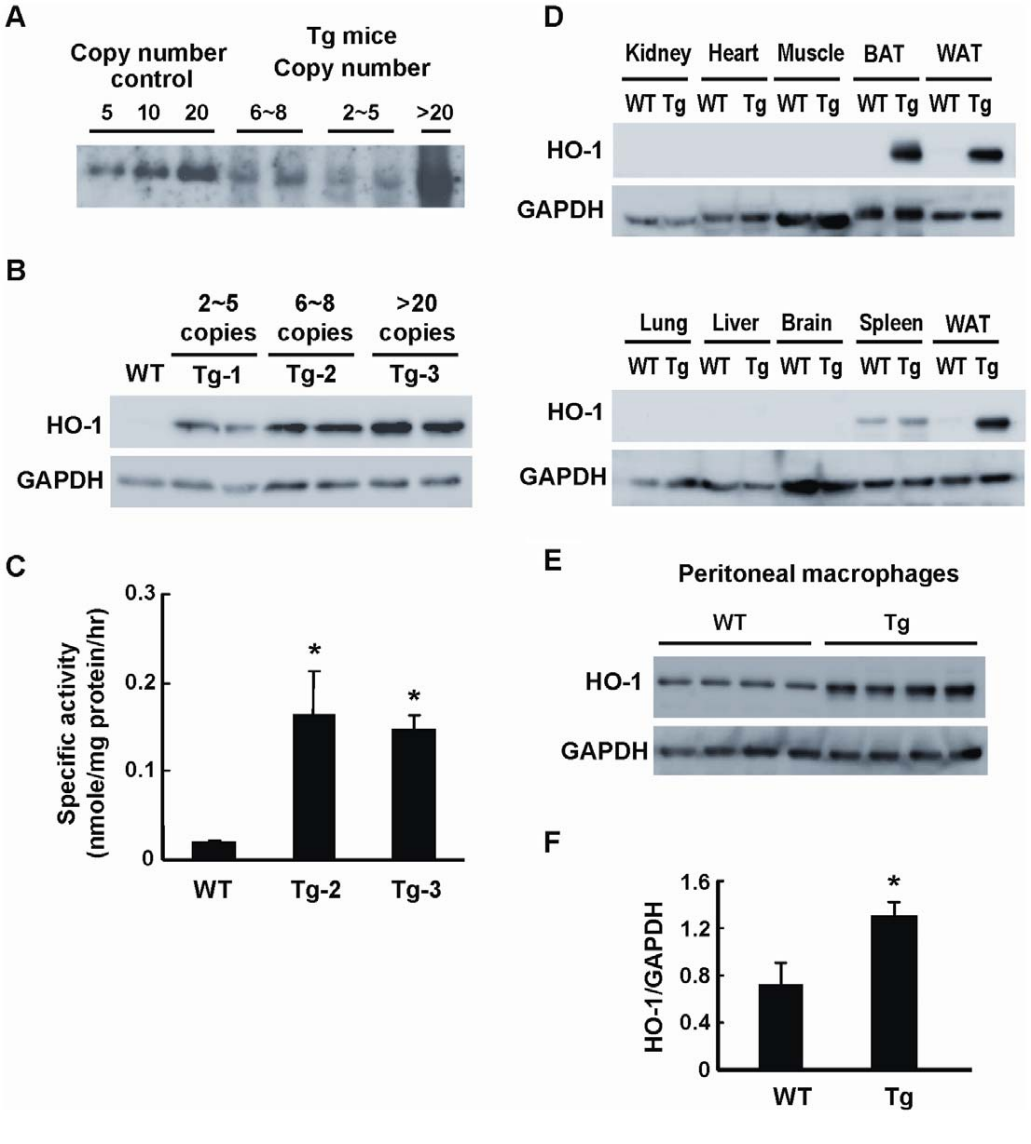
Generation of aP2-HO-1 Tg mice. A, Southern blot analysis of SacI-digested mouse genomic DNA from three lines of aP2-HO-1 Tg mice. The copy numbers of each line were estimated to be 2∼5, 6∼8 and >20 copies, respectively. B, HO-1 expressions in adipose tissues from three lines of aP2-HO-1 Tg mice were examined by Western blot analysis. C, Adipose HO-1 activities in WT and HO-1 Tg mice were determined. Data shown are means ± SE. *P<0.05 vs. WT. The number of mice in each group was four. D, HO-1 expressions in various tissues of WT and Tg mice examined by Western blot analysis. WAT, epididymal white adipose tissue; BAT, interscapular brown adipose tissue. E, HO-1 expressions in thioglycollate-induced peritoneal macrophages isolated from WT and Tg mice (n = 4/group) were examined. F, HO-1 protein levels of peritoneal macrophages were quantified by densitometry. *P<0.05 vs the WT group.

In an attempt to elucidate the effect of HO-1 on adipose remodeling and the development of insulin resistance during obesity, we used the adipocyte-specific aP2 promoter to generate Tg mice carrying human HO-1 gene. Three Tg mouse lines carrying various copy numbers of HO-1 gene were obtained (Fig. 1A). As assessed by Western blot analysis (Fig. 1B), the levels of HO-1 protein expression in epididymal WAT was significantly higher in the Tg mouse lines carrying 6∼8 (Tg-2) and >20 copies (Tg-3) of HO-1 gene as compared to that of WT control. To confirm that the HO-1 transgene is functionally active, we performed the HO enzymatic activity assay. As shown in Figure 1C, the HO activity of WAT was substantially higher in these two Tg mouse lines carrying the high copies of transgene. When HO-1 expressions in other tissues were examined, the results showed that HO-1 transgene overexpression was also noted in the interscapular brown fat tissue (BAT) of Tg mice (Fig. 1D). Whereas, HO-1 expressions in non-fat tissues, including heart, skeletal muscle, brain, liver, kidney, lung, did not show significant differences between WT and Tg mice (Fig. 1D). Since aP2 gene has been shown to be induced in activated macrophages [18]–[19], experiment was also performed to examine whether there is a leaked transgene expression in macrophages of Tg mice. As illustrated in Figure 1E and 1F, HO-1 protein expression was much higher in the peritoneal macrophages isolated from Tg mice as compared to WT counterparts.

### Tg Mice Exhibit a Metabolic Phenotype Similar to WT Mice

**Figure 2:**
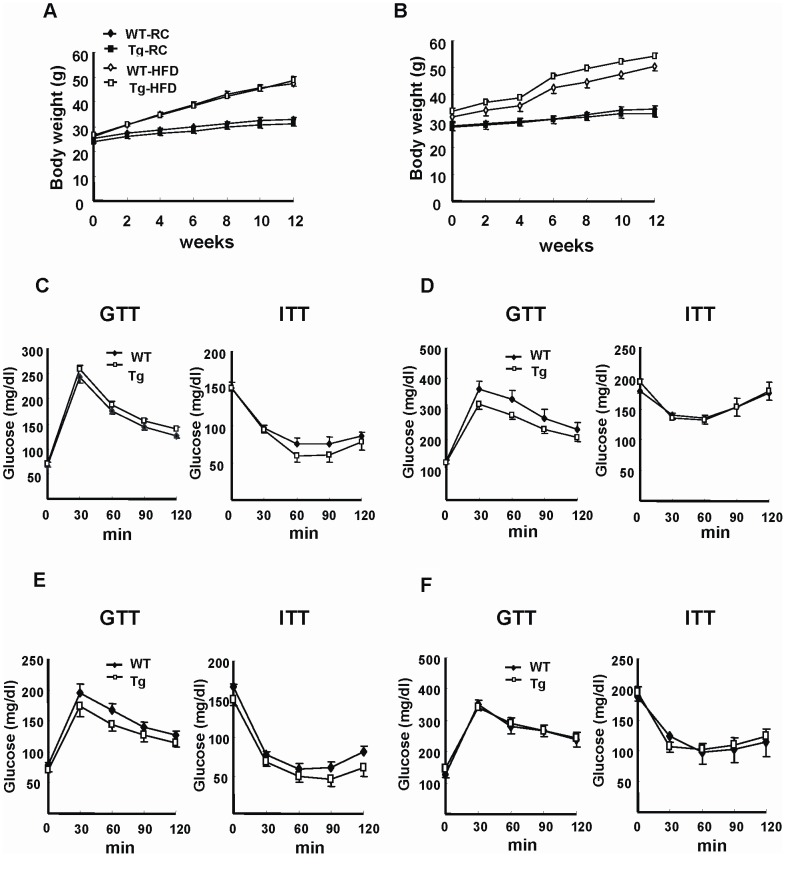
Tg mice exhibit similar metabolic phenotype as WT mice. A &B, HO-1 Tg (Tg-2 line, A; Tg-3 line, B) mice and their corresponding WT littermates were fed with a regular chow (RC) or HFD as indicated for 12 weeks. The body weights were measured every other week. The number of mice in each group was six to eight. C-F, WT and Tg mice (C&D,Tg-2 line; E&F, Tg-3 line) were fed with RC (C&E) or HFD (D&F) for 12 weeks, then the GTT and ITT assays were performed. The number of mice in each group was six to eight.

**Figure 3:**
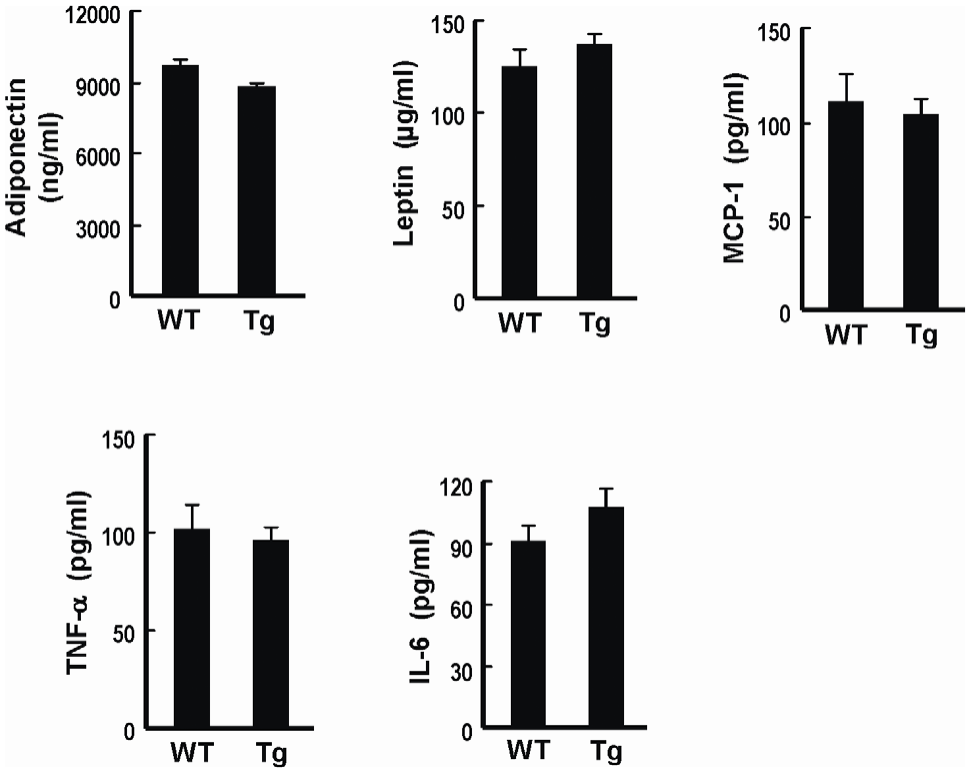
Serum adipokine levels in HFD-fed WT and Tg mice. Serum samples were collected from WT and Tg mice fed with HFD for 12 weeks, and the indicated adipokine levels were then determined by ELISA kits. The numbers of mice in WT and Tg groups were six and eight, respectively.

Figure 2A and 2B showed that the body weight gains of the Tg mice, Tg-2 and Tg-3 lines, respectively, were similar to their WT littermates when placed on regular chow (RC) diet for over 12 weeks. Likewise, food intake and serum concentrations of glucose, lipid, and insulin of Tg mice (Tg-3) and WT littermates were comparable (Table 1). Glucose tolerance test (GTT) and insulin tolerance test (GTT) again demonstrated no significant difference in insulin sensitivity between the lean Tg mice of either line and their WT counterparts (Fig. 2C and 2D). To investigate whether adipose HO-1 overexpression has an impact on diet-induced obesity and insulin resistance, Tg mice and their WT littermates were fed with HFD. As shown in Figure 2A and 2B, the body weight gains of both Tg mouse lines were similar to those of their respective WT controls. The serum glucose and insulin levels were significantly increased by HFD feeding to similar extents between the Tg and WT mice (Table 1). When these HFD-fed mice were subjected to GTT and ITT, the results again showed that both Tg lines of mice exhibited similar glucose intolerance and insulin resistance as compared to their WT counterparts (Fig. 2E and 2F). When the serum levels of adipokines, including adiponectin, leptin, IL-6, TNF-α, and MCP-1 in HFD-fed WT and Tg mice were determined, no significant difference was found between these two groups of mice (Fig. 3).

**Table 1:** Metabolic parameters of the WT and HO-1 Tg mice.

Diet	RC		HFD	
genotype	WT (n = 8)	Tg (n = 8)	WT (n = 6)	Tg (n = 8)
Body weight (g)	34.31±1.29	32.83±1.52	52.42±0.92^a^	55.95±1.24^a^
Food intake(g/d/mouse)	3.7±0.22	3.51±0.3	2.87±0.35^a^	2.87±0.14^a^
Liver (g)	1.36±0.08	1.31±0.07	2.43±0.34^a^	2.56±0.16^a^
eWAT (g)	1.75±0.2	1.36±0.21	4.51±0.43^a^	4.43±0.16^a^
Serum cholesterol(mg/dl)	108.25±4.29	99.25±1.63	230±13.73^a^	240.5±10.18^a^
Serum triglyceride(mg/dl)	66.5±5.18	88.5±8.54	66±6.02	57.88±2.52^a^
Serum glucose(mg/dl)	78.25±4.36	70.25±3.45	125.17±7.35^a^	144±8.49^a^
Serum insulin (ng/ml)	0.95±0.25	0.72±0.25	4.81±1.1^a^	6±2.13^a^

The number of animals in each group is indicated in parenthesis. Eight weeks old WT or Tg mice were fed with regular chow (RC) or high fat diet (HFD). At 12 weeks on the diets, serum samples were collected, and body weight, liver and epididymal white adipose tissue (eWAT) weights were determined. Data are expressed as the mean ± SEM.

a Significant difference between different diets within the same genotype.

### Effect of HO-1 on Adipose Gene Expression in Tg Mice

**Figure 4:**
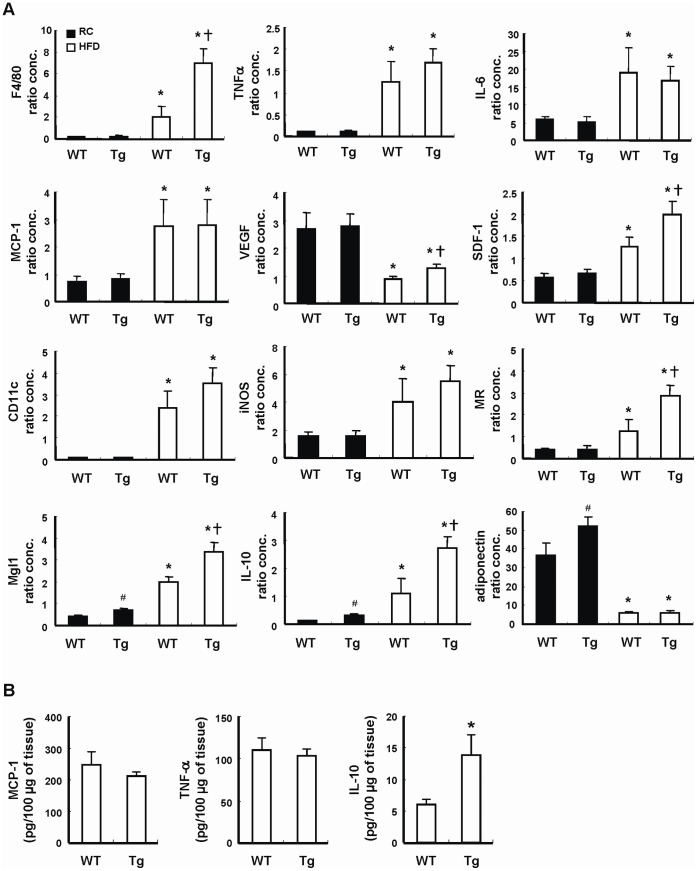
Adipokine gene expression in WT and Tg mice. WT and Tg mice were fed with a RC diet or HFD for 12 weeks, then the animals were killed and their visceral adipose tissues were collected. A, The expressions of the indicated genes in the adipose tissues were then analyzed by quantitative PCR. The expression level of 36B4 was used as an internal control for normalization. The number of mice in each group was six to eight. ^#^ *P*<0.05 vs RC-fed WT group; *P<0.05 vs the RC-fed group of the same genotype; ^†^ *P*<0.05 vs HFD-fed WT group. B, The levels of indicated adipokines in the WAT lysates of HFD-fed WT and Tg mice were determined by ELISA. The numbers of mice in WT and Tg groups were six and eight, respectively. *P<0.05 vs WT group.

HO-1 has been shown to modulate cytokine expression. To examine whether HO-1 overexpression in adipocytes affects adipokine gene expression, we performed quantitative real-time PCR with total RNAs isolated from the WAT of 20 weeks old male Tg mice (Tg-3) and the WT control placed on chow diet. As shown in Figure 4A, the adiponectin and IL-10 gene expression levels were significantly greater in WAT of lean Tg mice as compared to WT control. Nevertheless, the expression levels of TNF-α, IL-6, MCP-1, VEGF and SDF-1 were not significantly different between WT and Tg mice. Moreover, the gene expression levels of macrophage markers, including F4/80, CD11c, and iNOS, in lean WT and Tg mice were comparable as shown in the same figure. Nevertheless, the expression of Mgl1, a marker for M2 macrophages, was significantly higher in the adipose tissue of Tg mice as compared to WT controls, suggesting that adipose HO-1 overexpression may increase the polarization of adipose resident macrophages toward M2 phenotype even in the lean state. As demonstrated in the same figure, HFD caused a significant down regulation of adiponectin gene expression to a similar level in both WT and Tg mice. VEGF expression was also down-regulated in obese WAT, but its level was greater in obese Tg mice as compared to that of WT counterparts. The expressions of TNF-α, IL-6, IL-10, MCP-1, and SDF-1 in WAT were markedly induced by HFD. Interestingly, the levels of IL-10 and SDF-1 mRNAs induced were significantly higher in obese Tg mice as compared to WT controls. When the expression level of F4/80, an index of macrophage infiltration in WAT, was examined, the result showed that HFD-induced increase of F4/80 expression was much greater in Tg mice than WT controls. Although the expressions of CD11c and iNOs, the markers of M1 macrophages, were also significantly increased in both groups of mice after HFD feeding, the levels were not significantly different between these two groups. However, when the expression levels of MR and Mgl-1, the markers of M2 macrophages, were assessed, the data showed that their mRNAs were much higher in obese Tg mice than WT counterparts. Consistent with the mRNA expression levels, the levels of TNF-α, and MCP-1 proteins determined by ELISA did not show significant difference in the WAT of HFD-fed WT and Tg mice (Fig. 4B). However, the protein level of IL-10 was substantially greater in the WAT of HFD-fed Tg mice as compared to WT counterparts (Fig. 4B).

### HFD Promotes Angiogenesis and Macrophage Infiltration in WAT of Tg Mice

**Figure 5:**
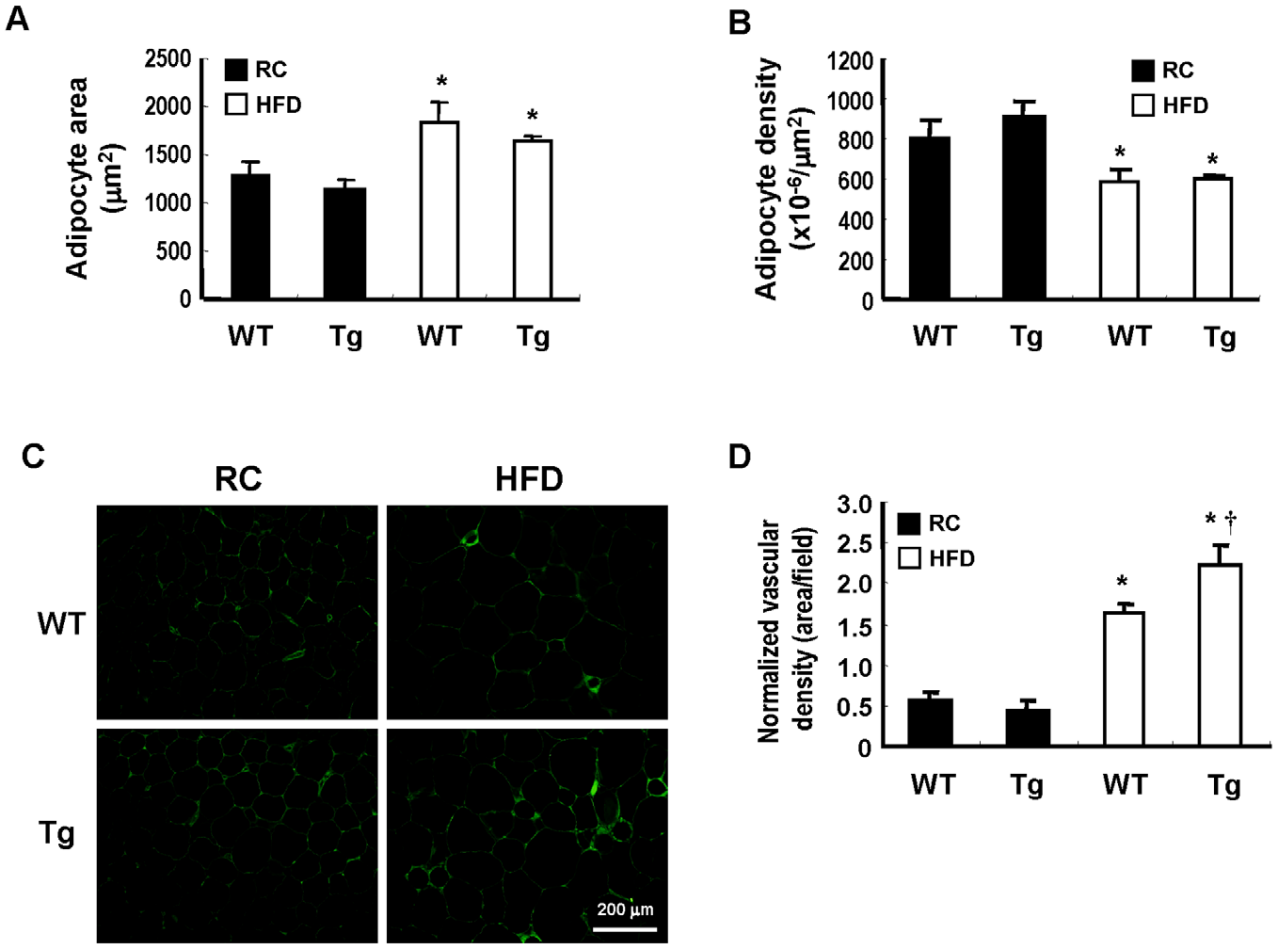
Effect of HFD on adipocyte hypertrophy and vascularization in visceral adipose tissues of WT and Tg mice. WT and Tg mice were fed with a RC or HFD for 12 weeks, and then sacrificed. A, The adipocyte size in the visceral adipose tissue sections of each group was analyzed. *P<0.05 vs the RC-fed WT group of the same genotype. B, The adipocyte density was calculated by counting the number of adipocyte per area. *P<0.05 vs the RC-fed group of the same genotype. C, The WAT sections were immunostained with antibody against CD31 as described in Materials and Methods. D, The normalized vascular density was quantified as the CD31-positve area divided by adipocyte density per filed. The number of animals in each group was five to six. *P<0.05 vs the RC-fed group of the same genotype; ^†^ *P*<0.05 vs HFD-fed WT group.

**Figure 6:**
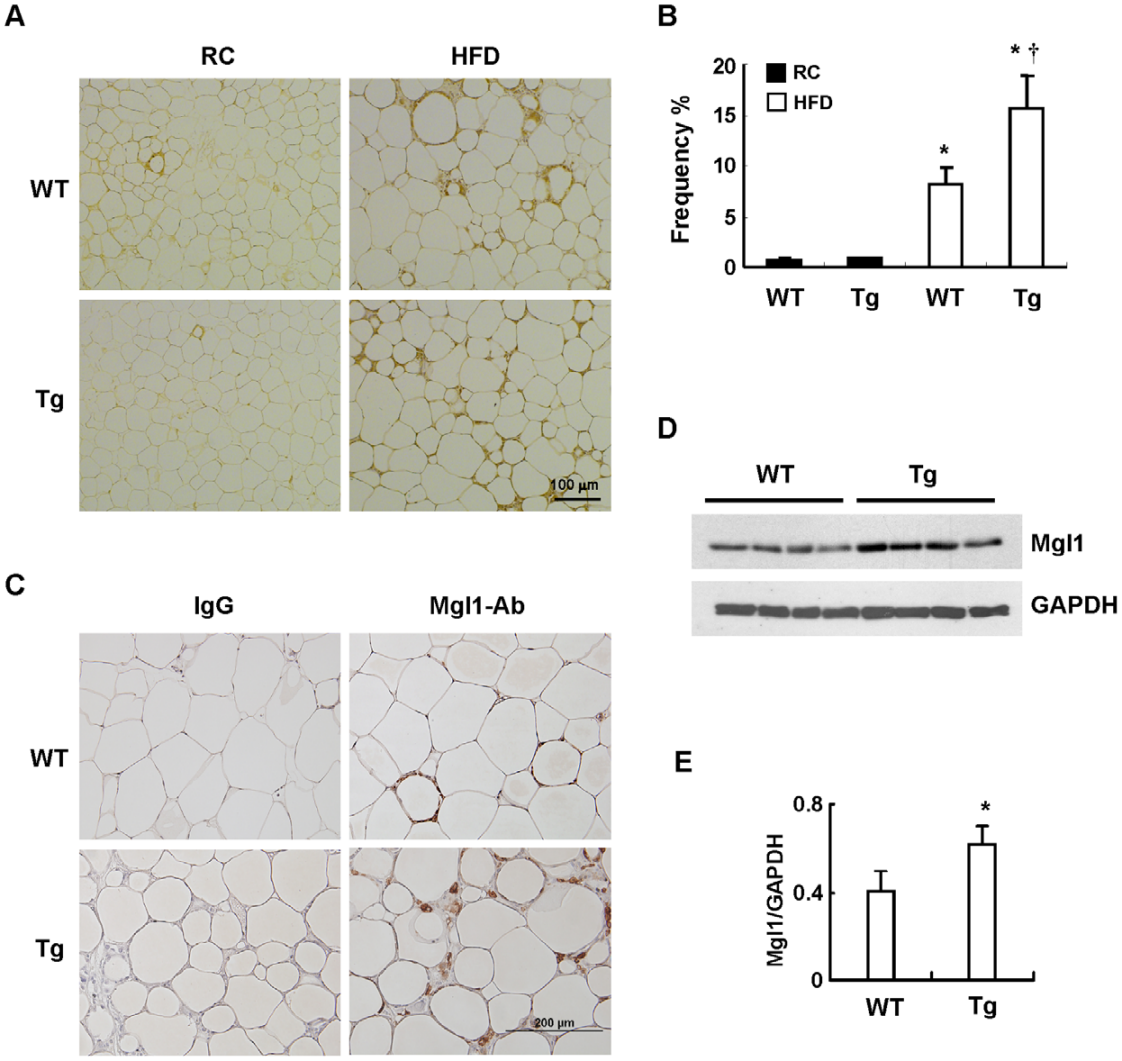
Effect of HFD on macrophage infiltration in visceral adipose tissues of WT and Tg mice. WT and Tg mice were fed with a RC or HFD for 12 weeks, and then sacrificed. A, The WAT sections were subjected to immunostaining using antibody against F4/80 as described in Materials and Methods. B, F4/80-positive macrophages were quantified as the percentage of adipocytes surrounded by a crown-like structure in the total adipocytes. The number of animals in each group was five to six. *P<0.05 vs the RC-fed group of the same genotype; ^†^ *P*<0.05 vs HFD-fed WT group. C, The WAT sections of HFD-fed WT and Tg mice were immunostained with antibody against Mgl1 as described in Materials and Methods. D, The Mgl1 expression levels in WAT tissues of HFD-fed WT and Tg mice were examined by Western blot analysis. E, The Mgl1 protein levels were quantified by densitometry. The number of animals in each group was four. *P<0.05 vs the WT group.

Histological examination of the visceral WAT of these mice after 12 weeks of HFD feeding revealed the enlargement of adipocytes and the decrease in the adipocyte density, which were not significantly different between WT and Tg groups (Fig. 5A and 5B). Immunostaining experiment performed using specific antibody against endothelial marker, CD31, demonstrated that the adipose vascular density was substantially higher in HFD-fed Tg mice as compared to that of WT counterparts. (Fig. 5C and 5D). When the immunostaining experiment was performed with F4/80 antibody, the results showed that the extent of macrophage infiltration, characterized by a crown-like structure surrounding dead adipocytes, in the WAT was significantly higher in HFD-fed Tg mice than in WT controls (Fig. 6A and 6B), which was consistent with the levels of F4/80 gene expression shown in Figure 4A. To examine whether the macrophages of HFD-fed Tg mice preferentially expressed M2 marker, Mgl1, we also performed immunostaining using antibody against Mgl1. As shown in Figure 6C, the positive immunostain for Mgl1 was more prominent in WAT of Tg mice than that of WT mice. Western blot analysis again demonstrated higher Mgl1 protein levels in the WAT lysates of Tg mice than WT counterparts (Fig. 6D and 6E).

### Myeloid Cells of Tg Mice Exhibit Greater Migration Response Toward Chemoattractant

**Figure 7:**
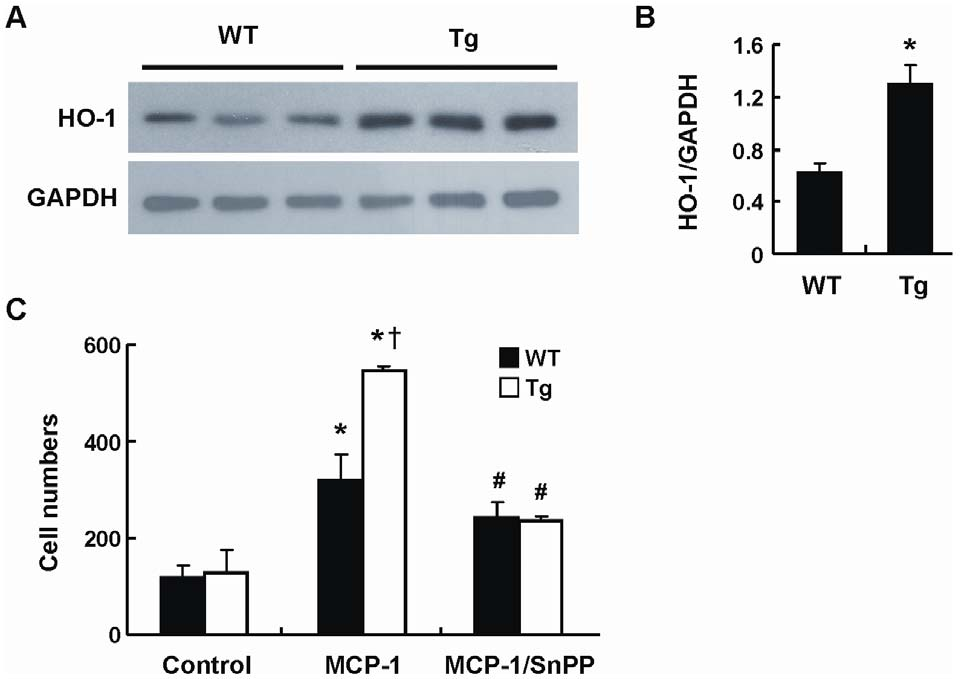
Myeloid cells of Tg mice exhibit greater migration response toward MCP-1. Splenic CD11b^+^ cells were isolated from WT or Tg mice. A, HO-1 expression level in each individual mouse was examined by Western blot analysis. B, HO-1 levels were quantified by densitometry (n = 3/group). *P<0.05 vs the WT group. C, CD11b^+^ cells isolated from each individual mouse were tested for their migration response toward 10 ng/ml of MCP-1 in the absence or presence of 10 µM SnPP. The number of mice in each group was three. *P<0.02 vs control group of the same genotype;^ †^ *P*<0.02 vs WT CD11b^+^ cells receiving MCP-1 treatment; ^#^ *P*<0.02 vs MCP-1-treated alone group of the same genotype.

We recently reported that HO-1 expression in monocytes/macrophages promotes chemoattractant-induced cell migration response [12]. To examine whether the increased adipose macrophage infiltration in HFD-fed Tg mice is due to the enhanced migration of myeloid cells to obese adipose tissue, we performed in vitro transwell migration assay using splenic CD11b^+^ myeloid cells isolated from Tg and WT mice. Similar to the peritoneal macrophages, CD11b^+^ cells of Tg mice expressed higher HO-1 than the WT counterparts (Fig. 7A and 7B). Although the basal migration response was comparable between WT and Tg myeloid cells, the migration response induced by MCP-1 was significantly greater in Tg group as compared to that of WT group (Fig. 7C). When cells were treated with 10 µM of SnPP, a HO inhibitor, MCP-1-induced migration response was markedly attenuated (Fig. 7C). Moreover, the difference in the migration response between cells of WT and Tg mice was abolished, supporting the role of HO-1 in the greater migration of myeloid cells of Tg mice.

## Discussion

Earlier studies have shown that chemical-induced systemic HO-1 overexpression inhibits obesity and improves insulin sensitivity in diabetic animals [13]–[15]. However, in the present study we found that the body weight or epidermal fat mass of lean Tg mice was not significantly different from that of WT mice. Although Tg mice (Tg-3 line) gained some more weight (∼6%) as compared to WT mice after HFD feeding, again the increases in the fat mass of both groups of mice were comparable. We reasoned that aP2 gene promoter is activated in the terminal differentiated adipocytes, therefore HO-1 transgene expression driven by aP2 gene promoter is not induced in the adipocyte progenitor cells in the Tg mice. In this case, HO-1 has no effect on the increased adipogenesis during HFD feeding in Tg mice. In subsequent experiment, we demonstrated that adipose overexpression of HO-1 did not provide salutary effect to protect mice from HFD-induced insulin resistance. We speculate that the discrepancy between our study and previous reports is caused by the differential involvement of tissues with HO-1 overexpression. In previous studies by others, chemical-induced HO-1 was not only expressed in adipocytes but also in non-adipose tissues. The effects of HO-1 on other tissues, such as liver and skeletal muscle, and their interplays with adipose tissue might contribute to the protective effect of HO-1 inducer observed in their experimental settings. However, more recently the same group reported that HFD caused down regulation of HO-1 in adipose tissue and the induction of HO-1 expression in adipocytes mediated by the intracardial injection of lentivirus bearing aP2-promoter driven human HO-1 gene was able to attenuate adiposity and vascular dysfunction in HFD-fed mice [20]. Again, their results are contradictory to the present findings with HO-1 Tg mice. The reasons for such discrepancy are unclear at the present time.

Histological examination of the adipose tissues revealed that the adipocyte hypertrophy observed in HFD-fed WT and Tg mice did not show significant difference. Whereas, immunohistochemical staining of endothelial cell marker, CD31, demonstrated that HFD-induced increase of adipose vascular density was much greater in Tg mice in the WAT of this group of mice. This observation was consistent with early works showing that HO-1 overexpression promotes angiogenesis [21]–[22]. When immunostaining experiment was performed with antibody against macrophage marker, F4/80, the results demonstrated that adipose macrophage infiltration was more prominent in HFD-fed Tg mice than WT mice. Likewise, quantitative PCR also confirmed the higher gene expression of F4/80 in HFD-fed mice as compared to WT counterparts. Nevertheless, when the expression profiles of adipokine genes linked to inflammation were analyzed, the results showed that the expressions of proinflmmatory cytokines, such as TNF-α and IL-6, in obese adipose tissues did not show significant difference between HFD-fed Tg and WT mice. To investigate the polarity of infiltrated macrophages, the relative expression levels of M1 and M2 macrophage markers were also assessed. The results showed that the expressions of CD11c and iNOS, the markers of M1 macrophages, were significantly induced by HFD in the adipose tissues of WT and Tg mice. However, the differences were not statistically different between two groups of mice. On the other hand, the increased expressions of MR and Mgl1, markers of M2 macrophages, were significantly greater in HFD-fed Tg mice as compared to WT mice. Immunostaining experiment and Western blot analysis again confirmed the higher expression of Mgl1 in WAT of HFD-fed Tg mice. These findings indicate that the macrophages recruited to obese adipose tissue of Tg mice are preferentially polarized toward M2 phenotype, which may explain why the higher degree of macrophage infiltration did not further augment adipose tissue inflammation in obese Tg mice. Notably, Mgl 1 expression was also much higher in the adipose tissue of lean Tg mice as compared to their WT counterparts, although the levels of F4/80 expression in both groups of mice were comparable. These observations suggest the possibility that HO-1 overexpression has an effect on the expression of adipocyte-derived Th2 cytokines, which are known to modulate macrophage alternative activation, in both lean and obese states [8] [23]. We paid particular interest in the expression of IL-10, which has been shown to be up-regulated by HO-1 [24] and has an effect to promote M2 polarization of adipose macrophages in obese animals [8]. As revealed by the gene expression analysis, IL-10 expression was significantly greater in the adipose tissue of Tg mice as compared to WT mice even in the lean state. HFD feeding led to markedly increase in adipose IL-10 gene expression in both WT and Tg mice. Again, both mRNA and protein levels of IL-10 expression induced by HFD was significantly greater in Tg mice than that in WT mice, suggesting that the higher content of M2 macrophages in the adipose tissue of obese Tg mice is likely mediated by the higher expression of IL-10.

Adiponectin is an insulin sensitizing adipokine specifically produced by adipocytes [6]. Its expression was inversely correlated with obesity and insulin resistance in experimental animals and human patients. It was found that adiponectin gene expression in lean Tg mice was significantly higher than the WT counterparts. This observation was consistent with previous reports showing that HO-1 increases adponection expression [13]–[15]. However, HFD feeding downregulated adiponectin expression in both WT and Tg mice to a similar level. Studies have shown that the inflammatory cytokines, such as TNF-α and IL-6, suppress adiponectin expression [6]. It is envisaged that the suppressive effect of inflammatory cytokines produced during obesity might surpass the promoting effect of HO-1 on adiponection gene expression in HFD-fed Tg mice. The failure to sustain high expression of adiponectin by HO-1 in obese state may contribute to the ineffective protection of Tg mice from the development of insulin resistance.

Although HO-1 transgene expression was primarily detected in the adipose tissues of Tg mice, we found a leaky expression of HO-1 transgene in myeloid cells in these mice. The expression of transgene in adipocytes and myeloid cells were also reported in other transgenic mice using the ap2 promoter [18]–[19]. Consistent with our early report showing that HO-1 promotes chemoattractant-induced myeloid cell migration [12], we demonstrated that myeloid cells isolated from Tg mice exhibited greater migration response toward MCP-1 than their WT counterparts. It is conceivable that the effect of HO-1 on myeloid cell migration may underlie the enhanced adipose macrophage infiltration in HFD-fed Tg mice. On the other hand, the expression levels of VEGF and SDF-1, but not MCP-1, in the adipose tissue of HFD-fed Tg mice were found to be much higher than their WT counterparts. As VEGF and SDF-1 are potent chemoattractants for myeloid cells [25]–[26], their high expression may also promote the recruitment of more monocytes/macrophages toward adipose tissue in obese Tg mice.

In conclusion, the present study demonstrated that HO-1 overexpression in adipocytes did not protect mice from obesity and the development of insulin resistance. HO-1 increased adiponectin expression in the adipose tissue of Tg mice in the lean state. However, it did not prevent the down regulation of adiponectin during obesity. Moreover, HO-1 transgene was also expressed in myeloid cells and likely promoted adipose macrophage infiltration in HFD-fed Tg mice. Although the adipose macrophages exhibit preferentially M2 phenotype in the obese Tg mice, they did not seem to improve the adipose inflammation state and the development of insulin resistance during obesity in Tg mice.
